# Annotations

**Published:** 1888-09-08

**Authors:** 


### ANNOTATIONS.

**The National Pension Fund and Its One Adverse Critic.**

THE *Lancet* has, as we anticipated, made another tilt at the National Pension Fund. This, however, is so feeble, and at the same time so ridiculous, as to require no detailed contradiction. It will be enough to point out one particular absurdity into which the *Lancet* has fallen, and this may be taken as a sample of the rest. Our contemporary enlarges upon the fact that a nurse of forty-nine next birthday must pay a premium of £20 18*s.* 8*d.* a-month for a pension of £15 a-year to be entered upon at the age of fifty; but it does not point out, what however everyone who has the most elementary knowledge of insurance can see, and what common sense would dictate, that a nurse of forty-nine entering upon a pension at fifty can only pay premiums for one year, whilst she may enjoy her pension for twenty or thirty years or more. In other words, the utmost possible amount that she can pay at £20 a month is about £250, whilst, supposing her to hold her pension for twenty-five years, which will often happen, she will receive altogether the sum of £375, together with her share of profits arising from surpluses and donations. This, the *Lancet* admits, may be a low rate of contribution, but the fallacy of the argument appears at once when our contemporary goes on to say that whilst one nurse will only be called upon to pay £272 2*s.* 8*d.*, another, within a few months of the same age, that is to say, just over forty-eight and not quite forty-nine, will be asked to pay £502 8*s.* This, however, is ridiculous, because when a nurse is asked to pay a premium of £20 a month, it is understood she will pay a definite number of premiums, and no more. So that whether a nurse be just over forty-eight or just under forty-nine she will pay the same number of premiums in each case. All this, as we have before said, is obvious to anyone who has the most elementary knowledge of insurance. This shows that the writer of the *Lancet's* article either had *not* the elementary knowledge, in which case he was not justified in writing at all, or, if he had, it proves that he was wilfully determined to do his utmost to throw dust into the nurses' eyes. A discussion carried on in such a manner is beneath contempt, and we merely allude to it to show into what desperate straits the *Lancet's* malignancy has led that once reputable journal. With regard to the references made to the new management, those who are best acquainted with the facts know that the insinuations are so futile and baseless as to be unworthy of comment.

**The Stable Door.**

THAT race of philosophers who think it the height of wisdom to lock the stable door after the horse is stolen is by no means extinct. There are many survivals both in town and country, and not a few are to be found among urban and rural sanitary authorities. The Local Government Board, by the mouth of its medical head, Dr. George Buchanan, F.R.S., has considered it desirable to address a few words of mild remonstrance to its local subordinates in the form of a "Memorandum," of which the main points are these: "English communities," says the memorandum, "nowadays recognise the advantage of isolation hospitals as a means of preventing the spread of infectious diseases from persons who cannot be properly isolated in their own homes. But too often the provision of such hospitals is put off until some infectious disease is immediately threatening or has actually invaded a district. It cannot be too clearly understood that an isolation hospital, to fulfil its proper purpose of sanitary defence, ought to be in readiness beforehand. During the progress of an epidemic it is of little avail to set about hospital construction. The mischief of allowing infection to spread from first cases will already have been done, and this mischief cannot be repaired. Thus hospitals provided during an epidemic are mainly of advantage to particular patients; they have little effect in staying the further spread of infection. Moreover, hospitals provided under such circumstances, to be of any use, must be large and costly; and their construction can seldom be of a kind that is suited in after times for the isolation requirements of their district." That is to say, a dam is built after the flood is past, and it is of such a kind that it will be of no use against future floods. How very obvious all this is to the most ordinary common sense. Nothing is more certain than that infectious diseases will spring up; nothing more certain than that if patients are not isolated at the outset the cases will be multiplied tenfold, to the danger and largely-increased cost of the community. It may be stated without question that an infectious diseases hospital provided after an outbreak of fever has commenced will generally require to be ten times larger than would be needed if the first few cases were promptly isolated. Therefore, though the building of a permanent hospital when the public health is good may seem an unnecessary expenditure, it will really prove in the long run an economy both of money and life. We commend the memorandum of the Local Government Board to the careful consideration of all local sanitary authorities.

**The Cancer Hospital at Brompton.**

DOWN in a corner of Brompton three hospitals dwell in amicable rivalry in good works. On one side of the street is the Hospital for Consumption and on the other is the Chelsea Hospital for Women and the Cancer Hospital. Sad stories in plenty might be heard within the walls of all three, but perhaps the largest proportion of really hopeless cases is to be found in the Cancer Hospital. The hospital itself is the lasting and beneficent memorial of a great sorrow. In 1840 Dr. Alexander Marsden lost his wife, who died of cancer after prolonged and severe suffering. This grief turned his attention to the study of the disease, and his efforts for the relief of it led to the foundation of the Brompton Cancer Hospital, which has now been enlarged to contain 120 beds. Of these about 108 are constantly occupied, and though the majority of the patients are women, it is noted that proportionately the disease is spreading more rapidly among men. Among the young and middle-aged patients—those under fifty years of age—the proportion of male to female patients is from a fourth to a sixth, but after that age things equalise themselves, till among the oldest cases men are in the majority. It is said, to the dismay of nicotians, that the spread of cancer among men is due to the habit of smoking. But it may be inferred that cancer is a constitutional or hereditary disease, from the fact that rather more than ten per cent. of the patients have relatives afflicted with diseases of a similar nature. The popular belief is that cancer is incurable, and there is no doubt that where the constitutional predisposition exists it is apt to recur; but if taken in time much can be done to eradicate the growths, or at worst to arrest the progress of the disease. It is true that there are sad sights to be met with in the Brompton Hospital—a girl of nineteen, with only three days of life before her, lying stupefied with morphia, given her to deaden her sufferings till death shall end them; or a young man condemned beyond the reprieve of the knife or the cautery to die of cancer of the liver. But there are other happier cases, where at least a temporary recovery has been won, and at the annual garden party, which is one of the chief festivals of the hospital, there is not a patient fit to move a limb who will not strain every nerve to present a cheerful face in the large and pleasant garden which surrounds the hospital like a visible emblem of the peace and ease that shelter the sufferers within.

